# Identification of a novel infection-enhancing epitope on dengue prM using a dengue cross-reacting monoclonal antibody

**DOI:** 10.1186/1471-2180-13-194

**Published:** 2013-08-29

**Authors:** Ya-Yan Luo, Jun-Jie Feng, Jun-Mei Zhou, Zhi-Zhun Yu, Dan-Yun Fang, Hui-Jun Yan, Gu-Cheng Zeng, Li-Fang Jiang

**Affiliations:** 1 Key Laboratory for Tropic Diseases Control, Ministry of Education of China, Department of Microbiology, Zhongshan School of Medicine, Sun Yat-sen University, Guangzhou 510080, China

**Keywords:** Dengue virus, prM protein, Epitope, Phage display peptide library, Antibody-dependent enhancement

## Abstract

**Background:**

Dengue virus (DENV) infection is the most important arthropod- borne viral disease in human, but antiviral therapy and approved vaccines remain unavailable due to antibody-dependent enhancement (ADE) phenomenon. Many studies showed that pre-membrane (prM)-specific antibodies do not efficiently neutralize DENV infection but potently promote ADE infection. However, most of the binding epitopes of these antibodies remain unknown.

**Results:**

In the present study, we characterized a DENV cross-reactive monoclonal antibody (mAb), 4D10, that neutralized poorly but potently enhanced infection of four standard DENV serotypes and immature DENV (imDENV) over a broad range of concentration. In addition, the epitope of 4D10 was successfully mapped to amino acid residues 14 to18 of DENV1-4 prM protein using a phage-displayed peptide library and comprehensive bioinformatics analysis. We found that the epitope was DENV serocomplex cross-reactive and showed to be highly immunogenic in Balb/c mice. Furthermore, antibody against epitope peptide PL10, like 4D10, showed broad cross-reactivity and weak neutralizing activtity with four standard DENV serotypes and imDENV but significantly promoted ADE infection. These results suggested 4D10 and anti-PL10 sera were infection-enhancing antibodies and PL10 was infection-enhancing epitope.

**Conclusions:**

We mapped the epitope of 4D10 to amino acid residues 14 to18 of DENV1-4 prM and found that this epitope was infection-enhancing. These findings may provide significant implications for future vaccine design and facilitate understanding the pathogenesis of DENV infection.

## Background

DENV is member of the genus *Flavivirus*. A sequence variation of 30% to 35% allows DENV to be divided into four related but antigenically distinct serotypes (DENV1-4). DENV represents a major arthropod-borne pathogen, leading to 390 million infections every year, mostly in the tropical and subtropical countries. DENV infection may cause a spectrum of clinical diseases, such as self-limited dengue fever (DF), potentially life-threatening dengue hemorrhagic fever (DHF) and dengue shock syndrome (DSS) [1]. In particular, the frequency of severe DENV infection in travelers visiting dengue endemic regions is similar to that of secondary infection in dengue endemic zones [2]. Although many studies have attempted to develop promising strategies, a specific antiviral agent to DENV infection or an approved vaccine remains unavailable [3,4].

The main obstacle to develop vaccines or specific antiviral therapies to DENV infection is that the immunopathogenesis of DENV infection is still not well known. Infection with one serotype can increase disease severity upon secondary infection with other serotypes. Additionally, infants born to dengue-immune mothers carries an increased risk of severe disease upon primary infection [5,6]. One explanation of severe DENV infections is the hypothesis of ADE [7]. According to this hypothesis, cross-reactive antibodies at sub-neutralizing concentrations generated during a primary infection has been suggested to enhance the subsequent infections by facilitating efficient binding and cell entry of virus-antibody complexes into Fc receptor-bearing cells [8]. Therefore, an effective dengue vaccine must provide a protective long-lasting immune response to all four serotypes; otherwise, vaccination itself could lead to additional risks. To date, clinical trials of dengue vaccine candidates did not show any ADE related to vaccination. However, it is necessary to ensure that infection enhancement will not occur in vaccinated population some time due to waning immunity [9]. Increased disease severity appears to associate with high viral load, suggesting that antibodies have an influence on the infectious properties of the virus. The viral load in secondary infection patients without severe disease is similar to that in primary infection patients [10]. However, it has been reported that pre-existing antibody in infants did not correlate with increased viremia and disease severity [11]. In addition, it has also been demonstrated asymptomatic healthy blood donors with high dengue viremia did not have detectable antibody [12]. To date, understanding of the molecular mechanism of ADE has been severely hampered by the lack of an ideal animal model and is still a mystery. Consequently, there have been few reports to provide in vivo evidence of ADE of DENV infection [13,14]. In spite of that, it has been proved that AG129 mouse model could cause many similarities with specific features of human DENV infection and thus may be an appropriate animal model for the investigation of ADE in vivo [15].

DENV is an enveloped, positive-stranded RNA virus and it encodes three structural proteins (capsid, C; envelope, E; pre-membrane, prM) and seven nonstructural (NS) proteins (NS1, NS2a, NS2b, NS3, NS4a, NS4b and NS5) [16]. The initial assembly of DENV particles occurs in the endoplasmic reticulum by formation of immature virions, which contain heterodimers of the E and prM. The prM protein is a 166-amino-acid protein, which is believed to acts as a chaperone for correct folding and assembly of the E protein [17,18]. Subsequently, the viral envelope proteins are believed to undergo conformational changes triggered by the low pH in trans-Golgi network (TGN). Then the endoprotease furin cleaves prM into M that remains associated with the virus particle and an N-terminal 91-amino-acid peptide (“pr”) that dissociates upon release of the virus from the infected cell, resulting in the formation of mature virions [19,20]. The cleavage of prM to M is required for DENV maturation and infectivity.

Many studies have shown that prM-specific antibodies could mediate DENV-specific immune response in humans [21-25]. These prM-specific mAbs are highly cross-reactive among four DENV serotypes and, even at high concentrations, do not neutralize infection but potently promote ADE infection over a broad range of concentration [24,26]. It has also been suggested that anti-prM antibodies could render essentially non-infectious imDENV highly infectious [24,27,28]. Recent studies in infants have also implicated that anti-prM antibodies could lead to severe disease upon primary infection [29]. These studies on human and mouse anti- prM mAbs [30-32] suggest that this class of antibodies have a significant role to enhance DENV infection in humans. However, most of the epitopes recognized by these prM-specific antibodies remain unclear.

Viral protein epitopes are pivotal in the pathogenesis of virus infection and in the development of effective vaccines [33,34]. Therefore, the identification of B-cell epitopes for DENV prM antibodies can provide important information for the understanding of the pathogenesis of DENV infection and contribute to the development of dengue vaccine. In the case of DENV, many efforts have been made into mapping the epitopes of E protein [35-39], but only a few epitopes have been identified on prM protein [40,41]. Consequently, the precise antigenic structures of prM and their functions in the immune response and infection pathogenesis remain poorly studied.

In the present study, the epitope recognized by prM mAb 4D10 was identified using a phage-displayed peptide library and comprehensive bioinformatic analysis. We investigated the neutralizing versus enhancing capacity of the mAb 4D10 and antisera of epitope peptide PL10 towards standard DENV1-4 particles and imDENV particles. We found that 4D10 and antibody against epitope peptide PL10 showed broad cross-reactivity and poor neutralizing acvitity with the four standard DENV serotypes but significantly enhanced the infectious properties. In addition, these antibodies remained susceptible to partially neutralizing imDENV and indeed rendered virtually non-infectious imDENV highly infectious in Fc receptor-bearing cells. Taken together, we identified a novel infection-enhancing epitope on prM protein. These results may provide some important implications for a better understanding of the pathogenesis of DENV infection and advance the development of dengue vaccine.

## Methods

### Cells

C6/36 cells derived from *Aedes albopictus* were maintained in Modified Essential Medium (GIBCO) supplemented with 10% fetal bovine serum (FBS) at 28°C, 5%CO2. Baby Hamster Kidney-21 (BHK-21) cells derived from the kidney of *Mesocricetus auratus* and Human adenocarcinoma LoVo cells derived from left supraclavicular region metastasis were cultured in Dulbecco’s Modified Eagle’s Medium (GIBCO) supplemented with 10% FBS at 37°C, 5% CO2. Human erythroleukemic K562 cells derived from bone marrow were maintained in Iscove’s Modified Dulbecco’s Medium (GIBCO) supplemented with 10% FBS at 37°C, 5% CO2. The media were supplemented with 2 mM L-glutamine, 10mM HEPES, penicillin (100 U/ml) and streptomycin (100 U/ml). All cells were purchased from ATCC.

### Viruses

DENV1 strain Hawaii (GenBank: EU848545), DENV2 strain New Guinea C (NGC) (GenBank: AF038403), DENV3 strain H87 (GenBank: M93130), DENV4 strain H241 (GenBank: AY947539) and JEV (GenBank: AF315119) were propagated on C6/36 cells. Briefly, monolayer of C6/36 cells was infected with DENV at multiplicity of infection (MOI) of 1. The virus supernatants were harvested at 72 hours post-infection (hpi), cleared from cellular debris by low-speed centrifugation, purified by PEG 8000 precipitation. Fully imDEVN2 NGC strain was produced on furin-deficient LoVo cells as described before [42]. Briefly, LoVo cells were infected at MOI 10 for 1.5h at 37°C. Then, virus inoculum was removed and fresh medium was added after washing the cells twice with PBS. At 72 hpi, the virus particles were harvested,cleared from cellular debris by low-speed centrifugation. Subsequently, virus particles were precipitated by 40% PEG 8000. The titers of virus were determined by plaque assay on BHK-21 cells and viral RNA copy numbers were calculated by real-time quantitative RT-PCR (qRT-PCR). To assess the growth and infectious properties of standard DENV2 and imDENV2 at different time point, standard DENV2 and imDENV2 were cultured in C6/36 cells and LoVo cells respectively at MOI 10 and virus particles were collected at 24 h time intervals (24 hpi, 48 hpi, 72 hpi, 96 hpi).

### Antibodies

2H2 (IgG2a anti-DENV1-4 prM) and 4G2 (IgG2a anti-all flavivirus E) hybridomas were purchased from ATCC. 4D10 (IgG1 anti-DENV1-4 prM) hybridoma was generated according to standard procedures [43]. Briefly, Six-week-old female BALB/c mice were subcutaneously immunized twice at 2-week intervals with purified prM in Freund’s complete or incomplete adjuvant (Sigma). Three days after a final immunization, spleen cells from the mice and mouse myeloma SP2/0 cells were fused and maintained according to the standard procedure [43]. The hybridoma producing 4D10 (IgG1) was screened by enzyme-linked immunosorbent assay (ELISA), western blot analysis and indirect immunofluorescence assay (IFA). 4D10 (IgG1) was purified from mouse ascites using protein A affinity columns (GE).

### Human serum samples

Human serum samples were obtained from DENV2 patients or healthy adults after consent and approvals from the ethical committee of Haizhu district center for disease control and prevention of Guangzhou, China. The study was also approved by the Animal Experimentation Ethics Committee of Sun Yat-sen University. Acute DENV2 infection was identified by virus isolation during C6/36 cell culture and DENV serotype-specific reverse transcriptase-PCR (RT-PCR) [44]. DENV infection was also confirmed by DENV-specific IgG and IgM capture ELISA [45].

### Phage-displayed biopanning procedures

The Ph.D.-12™ Phage Display Peptide Library Kit was purchased from New BioLabs Inc. Four successive rounds of biopanning were carried out according to the manufacturer’s instruction manual. Briefly, 100 μl mAb 4D10(100 μg/ml) was coated overnight at 4°C on 96-well plate and blocked at 4°C for 2h. The plates were then washed five times with washing buffer, and phages [1.5×10^11^ plaque-forming units (PFU)] were incubated at 37°C for 1h with coated antibody. The wells were washed five times with TBST. Then, the bound phages were eluted with 100 μl of 0.2 M glycine-HCl (pH 2.2) plus 1 mg of BSA/ml and were then neutralized with 15 μl of 1 M Tris–HCl (pH 9.1). The eluted phages were amplified and titrated in Escherichia coli ER2537 culture. The amplified phages were used in the next cycle. After four rounds of biopanning, 62 phage clones were selected and screened by ELISA and further characterized by DNA sequencing.

### ELISA

To identify immunopositive phage clones, the ELISA plates were coated overnight at 4°C with 100 μl mAb 4D10(100 μg/ml) and blocked 2h at 4°C. Phage clones were added to the wells (1.5 × 10^11^ pfu in 100 μl per well) and incubated with agitation for 2h at room temperature. The plates were then washed with washing buffer, and 1:5000-diluted horseradish-peroxidase (HRP)-conjugated anti-M13 antibody (Pharmacia) in blocking buffer was added. The plates were incubated at room temperature for 1 h with agitation and washed with washing buffer. HRP/substrate solution was added to each well and incubated at room temperature. The reaction was stopped with 2 N H_2_SO_4_ and the plates were read using a microplate reader set at 450 nm.

For antibody-binding assay, ELISA plates were coated with 100 μl per well of individual synthetic peptides at a concentration of 10 μg/ml. For the sensitivity binding assay, 2-fold serial peptide antigens (concentrations ranging from 20 to 0.31 μg/ml) were coated to the plates. Anti-prM mAb diluted in 1:200 was added to each well. Subsequently, the wells were incubated with corresponding HRP-conjugated anti-mouse IgG, then the same steps as above were followed and absorbance was measured.

### DNA sequencing and computer analysis

The DNA sequences of ELISA-positive phage clones were sequenced with the 96 gIII sequencing primer: 5’-TGAGCGGATAACAATTTCAC-3’, based on phage cloning vector (GenBank: L08821), as described by the manufacturer’s instructions (New England BioLabs Inc.). Sequences of DNA inserted into target phage clones were translated into amino acid sequences and aligned with that of prM protein of DENV2 using Standard protein–protein BLAST [blast] and ClustalW Multiple Sequence Alignment [clustal] public software.

### Bioinformatics analysis of DENV2 prM B-cell epitopes

Using DNASTAR software and ExPaSy multiple bioinformation software, we performed general evaluation of DENV prM B-cell epitopes including Hopp &Wood hydrophilicity; Granthan polarity; Jameson & Wolf antigenicity; Bhaskaran & Ponnuswamy flexibility; Emini accessibility; Deleage & Roux alpha-helix regions; Deleage & Roux beta-turn regions [46-51]. Considering the results of phage biopanning together, one predominant epitope peptide PL10 (^13^IVSRQEKGKS^22^) (GenBank: AAC59275), control peptides PH10 (^3^LTTRGGEP HM^12^) (GenBank: AAC59275) and PM10 (SQNPPHRHQS) (Ph.D.-12™ Phage Display Peptide Library Kit, New BioLabs Inc.) were synthesized (purity >95%, China Peptides Co., Ltd).

### Competitive-inhibition assay

In competitive-inhibition experiments, coating with anti-prM mAb, blocking, and washing were performed. Synthetic peptide PL10 was added 0.1 μg per well and corresponding phage clones were added simultaneously. Then the same steps as described in “ELISA” were followed. The inhibition percentage was calculated as follows: inhibition (%) = [(OD450 without competitor −OD450 with competitor)/ OD450 without competitor] ×100%.

### Immunization assay and protection assay in adult Balb/c mice

All procedures involving animals were approved by the Animal Experimentation Ethics Committee of Sun Yat-sen University and carried out by a licensed individual with an ethical approval number of 2012/0081.

Animals were purchased from the Center of Experimental Animal of the Sun Yat-Sen University. Four groups (PL10 coupled to KLH, PH10 coupled to KLH, PM10 coupled to KLH, and PBS), each comprising of ten adult female Balb/c mice (4–6weeks old), were intraperitoneally injected with 100 μg of immunogen emulsified in complete Freund’s adjuvant for the first immunization. Mice were then injected at week 2 and 4 with the peptides and Freund’s incomplete adjuvant. The mice were bled on week 0, 2, 4 and 6 via tail vein according to NC3Rs standard procedures, and the anti-peptide antibody titer of mice sera was determined by ELISA.

Two weeks after the last immunization, mice were infected with DENV2 NGC strain (10^6^ PFU/mouse) through peritoneal injection. Blood samples were collected at day 0.25, 1, 2, 3, 4 and 5 via tail vein according to NC3Rs standard procedures. Then, all animals were euthanized by using Carbon dioxide (CO_2_) according to NC3Rs standard procedures and the experiment was terminated. Viral RNA was extracted from 140 μl serum aliquots using QIAamp Viral RNA mini kit (Qiagen). The viral RNA copy numbers were quantified by qRT-PCR.

### Western blot analysis

DENV infected C6/36 cells were treated with 1% triton X-100, the lysates were run on 12% SDS polyacryramide gels and transferred onto polyvinylidene difluoride (PVDF) membranes (Amersham). The membranes were then blocked with PBS containing 5% skimmed milk and probed with prM-specific antibodies for 2 h at room temperature. Subsequently, membranes were detected with HRP-conjugated anti-mouse IgG and developed with enhanced chemiluminescence reagents (ECL, Thermo Fisher Scientific).

### Indirect immunofluorescence assay (IFA)

C6/36 cells were infected with DENV1-4 and JEV. Cells were then fixed with acetone at −20°C for 20 min and washed three times with PBS. Cells were incubated with a 100-fold dilution of prM-specific antibodies. After 60 min of incubation at 37°C, cells were washed three times with PBS. Cells were then reacted with a 200-fold dilution of Alexa-Fluor-488-conjugated anti–mouse IgG (Invitrogen) for 45 min at 37°C, washed five times with PBS. After washing, cells were treated with DAPI and detected using a fluorescent microscope.

### Real-time quantitative RT-PCR (qRT-PCR)

Viral RNA copy numbers were quantified by qRT-PCR as described previously [52]. Briefly, Viral RNA was extracted from 140 μl serum aliquots using QIAamp Viral RNA mini kit (Qiagen). DENV1-4 serotypes universal primers (Forward Primer: 5'-GCATATTGACGCTGGGAGAGA-3', Reverse Primer: 5'-GCGTTCTGT GCCTGGAATG-3') and Probe (5'-FAM-AGATCCTGCTGTCTCTACAMGB-3'), based on 3' noncoding region of DENV1 strain Hawaii (GenBank: EU848545), were selected by using Primer Express software. cDNA was synthesized from viral RNA ( 37°C for 15 min, 85°C for 5 s) by using PrimeScript® RT reagent Kit (Takara). DNA was amplified for 40 cycles (95°C for 10 s, 95°C for 5 s, 60°C for 20 s) by using Premix Ex Taq™ (Takara). The concentration of viral RNA copy numbers was determined using a standard curve based on a cDNA plasmid containing the gene fragment of 349 bp in 3' noncoding region of DENV1 strain Hawaii.

### Plaque forming assay

The titres of virus were determined by a plaque forming assay on BHK-21 cells and expressed as PFU per ml. Briefly, virus was serially 10-fold diluted and incubated with BHK-21 cell monopayers for 2h at 37°C. The monolayers were then overlaid with 1.2% (w/v) carboxymethylcellulose and incubated at 37°C for 7 days. The wells were stained with 1% (w/v) crystal violet dissolved in 4% (v/v) formaldehyde to visualize the plaques. Plaques were counted and the virus titer was expressed as PFU/ml.

### Plaque reduction neutralization test (PRNT)

Neutralizing activity of prM-specific antibodies was determined by the plaque reduction neutralization test (PRNT). Briefly, 2-fold serially diluted antibody was mixed with approximately 50 PFU DENV and incubated for 1h at 37°C. The mixtures were then transferred to BHK-21 cell monolayers followed by the plaque forming assay described above. The percentage of plaque reduction was calculated as previously described [53].

### Antibody-dependent infection enhancement assay

Serial 2-fold dilutions of antibody were incubated with an equal volume of DENV for 1h at 37°C then transferred to K562 cells at MOI of 1and incubated at 37°C for 4 days. Supernatants were then harvested and viral RNA levels were assessed by qRT-PCR as described above. Alternatively, after 3 days, infected K562 cells were determined by flow cytometry.

### Flow cytometry

The infected K562 cells were fixed and permeabilised with Cytofix/ Cytoperm™ Fixation/Permeabilization kit (BD) at 4°C for 20 min. DENV antigens were then stained with 4G2 conjugated to Alexa-Fluor-488 (Invitrogen) at 4°C for 30 min. The cells were washed twice, and percent infection was determined by flow cytometry.

### Statistical analysis

Statistical analyses were performed in GraphPad Prism 5.0 software. ANOVA Tukey’s post-hoc statistical tests were used for pairwise comparisons of multiple groups. A p value of less than 0.05 was considered significant.

## Results

### Characterization of DENV-specific mAb 4D10

**Figure 1:**
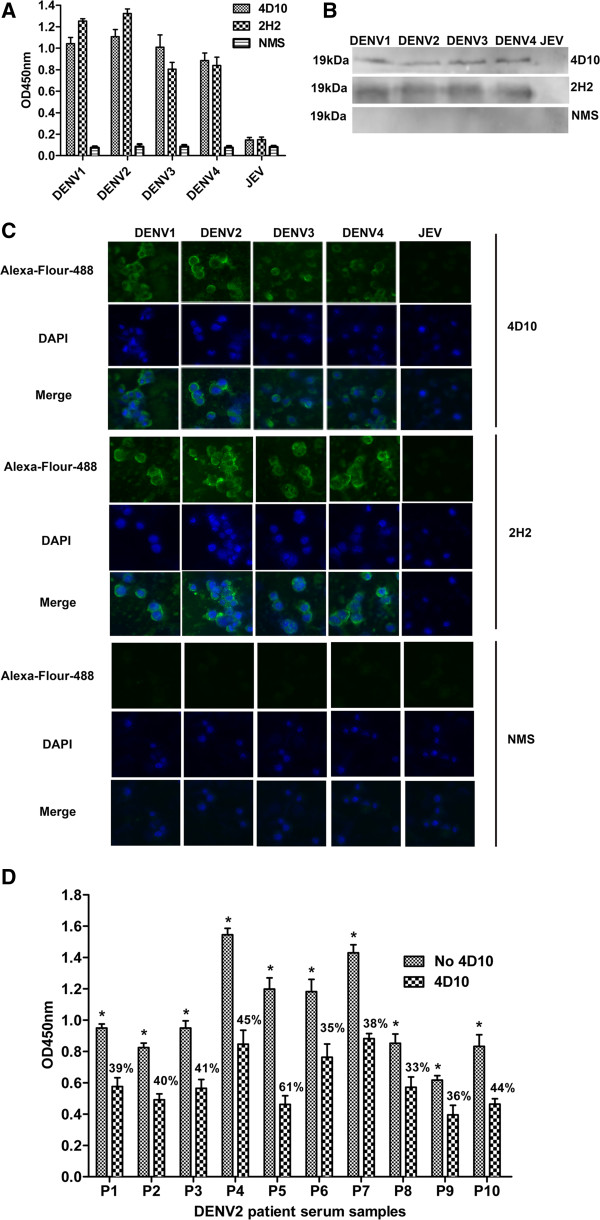
**Characterization of prM mAb 4D10. (A, B** and **C)** Cross-reactivity of 4D10 with four DENV serotypes and JEV (negative control antigen for the specificity of the antibody 4D10) determined by ELISA **(A)**, western blot **(B)** and IFA **(C)**. These results showed that only DENV1-4 infected C6/36 cells could be detected with 4D10 and 2H2 (positive control antibody) but not JEV infected cells. Normal mouse serum (NMS) had no such reactivity with all flaviviruses. **(D)** Competitive inhibition of DENV2 patient sera binding to DENV2 by mAb 4D10. Competitive ELISA was performed using 4D10 as competitor of DENV2 patient sera. The percentage of inhibition is also shown. Data are expressed as means of at least three independent experiments. The error bars represent standard deviations (SD). If there is no error bar, it is not that no variations among three independent experiments but that the variations are too small to show in the figure. * *P* < 0.05 vs 4D10.

ELISA assays showed that 4D10, like 2H2 (positive control antibody), detected DENV1-4 infected cells but not JEV (negative control antigen for the specificity of the antibody 4D10) infected cells (Figure 1A). Western blot analysis confirmed that the specificity of 4D10 and 2H2 for DENV1-4 prM protein (Figure 1B). To further prove the DENV serotypes specificity of 4D10, we also performed an indirect immunofluorescence assay (Figure 1C). Only DENV infected C6/36 cells were detected by 4D10 and 2H2, JEV infected cells were not reactive. These results suggested that 4D10 is similar to 2H2, which has been proved to be a DENV cross-reacting prM mAb [40]. We concluded that 4D10 is a DENV serocomplex cross-reactive prM mAb that does not cross-react with other flaviviruses.

To confirm further the specificity reactivity of 4D10, an antibody competitive- inhibition assay was carried out to determine whether the 4D10 competed with DENV2 patient sera for reactivity with DENV2. The reaction activity of DENV2 patient sera with DENV2 was inhibited markedly by 4D10 with the inhibition percentage from 33% to 61% (Figure 1D).

### Screening of phage-displayed peptide library with anti-DENV prM mAb 4D10

**Figure 2:**
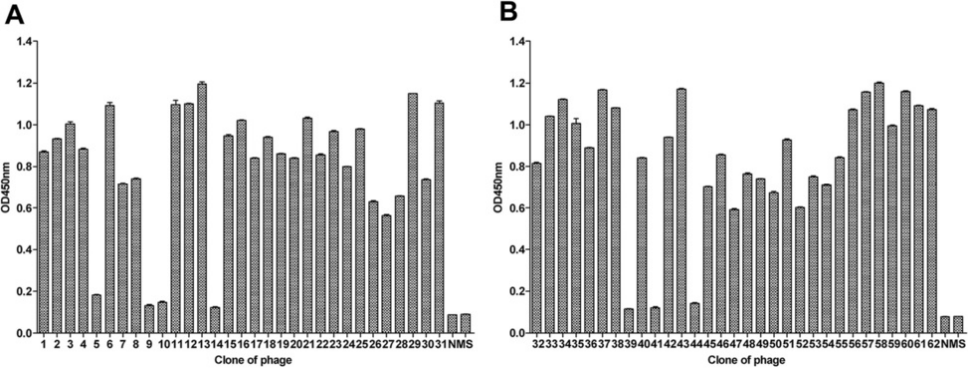
**Selection for specific phage clones bound to mAb 4D10. (A)** Twenty-seven phage clones reacted strongly with 4D10. **(B)** Twenty-eight phage clones reacted strongly with 4D10.After the fourth round of biopanning, 55 phage clones from 62 selected phage clones showed significant reactivity to mAb 4D10 but not to normal mouse serum (NMS). Data are expressed as means of at least three independent experiments. The error bars represent standard deviations (SD). If there is no error bar, it is not that no variations among three independent experiments but that the variations are too small to show in the figure.

To select the immunopositive phage clones, anti-DENV1-4 prM mAb (4D10) was purified from the ascites using the protein A affinity column. The bound phage clones were selected after four biopanning rounds. Fifty-five of 62 selected phage clones had significant enhancement of reactivity to mAb 4D10 but not to normal mouse serum (NMS) (Figure 2). Inserted nucleotides of the selected positive phage clones were sequenced and translated to peptide sequences (Table 1). Through alignment of phage-displayed peptide sequences using DNASTAR software, the binding motif of antibody 4D10 was shown to be VS/GKTE (Table 1). We next compared the binding motif with the primary amino acid sequence of the prM protein of DENV1-4, YFV, WNV, JEV and TBEV and found that the epitope for antibody 4D10 corresponded only to amino acid residues 14 to18 of DENV1-4 prM protein but not to other flaviviruses (Table 2). Notably, the epitope for antibody 4D10 is only conserved among four DENV serotypes.

**Table 1:** Amino acid sequence analysis of selected phages screened against prM mAb 4D10

**Peptide name/frequency**	**Peptide sequence**
P1 (24)	T**VSK**T**E**SLYRPW
P2 (21)	T**VSK**T**E**LLYRPR
P3 (1)	S**VGK**T**E**SLYRPW
P4 (5)	T**VSK**T**E**SPYRPW
P5 (1)	AVEQEAARHYNW
P6 (2)	HSPYWLIQASRQ
P7 (1)	MVSQNPPHRHQS
Consensus	**VS/GK**T**E**

Notes: Phage-displayed consensus amino acids are shown in bold.

**Table 2:** Alignment of amino acid residues 14 to 18 of the prM proteins of flaviviruses with binding motif VS/GKTE

**Virus**^**a**^	**Amino acid sequence**
Binding motif	**VS/GK**T**E**
DENV1	I**VSK**Q**E**RGKSLL
DENV2	I**VSR**Q**E**KGKSLL
DENV3	I**VGK**N**E**RGKSLL
DENV4	I**V**A**K**H**E**RGRPLL
WNV	TVNATDVTDVIT
JEV	TINNTDIADVIV
YFV	NVTSEDLGKTFS
TBEV	AEGKDAATQVRV

*Notes*: ^a^The protein sequences of DENV1, DENV2, DENV3, DENV4, WNV, JEV, YFV and TBEV were retrieved from GenBank with accession numbers EU848545, AF038403, M93130, AY947539, DQ211652, AF315119, X03700 and AY182009 respectively. The amino acids identical between the binding motif and prM protein are shown in bold.

### General evaluation of DENV prM epitopes with bioinformation software

In order to select the predominant epitopes of DENV prM, we performed general evaluation of DENV prM protein sequence including Hopp & Wood hydrophilicity; Granthan polarity; Jameson & Wolf antigenicity; Bhaskaran & Ponnuswamy flexibility; Emini accessibility; Deleage & Roux alpha-helix regions and beta-turn regions. The epitopes are most likely fall on the regions that have shown in Table 3. According to the empirical rules that the positions of B-cell epitopes ought to be located at the region which contained more beta-turns but fewer alpha-helixes, as well as be hydrophilic, polar, antigenic, flexible, and accessible, we found that one of possible B-cell epitopes was located in amino acid residuals 12–26 (Table 3).

**Table 3:** Prediction of B-cell epitopes of DENV prM protein

**Predicted criteria**	**B epitope regions**
Hopp & Wood hydrophilicity	5–10, **12–26,** 42–47, 56–66, 83–94, 102–112, 115–122
Granthan polarity	5–9, **15–20,** 58–63, 83–91, 116–118
Jameson & Wolf antigenicity	3–12, **14–24,** 26–33, 40–53, 56–73, 81–94, 111–118, 130–133
Bhaskara & Ponnuswamy flexibility	5–9, **15–20,** 55–66, 85–91, 103–106, 108–118
Emini accessibility	3–9, **15–21,** 24–29, 47–50, 56–62, 82–92, 104–110, 119–124
Deleage & Roux alpha-helix regions	5–12, **16–19,** 23–34, 44–58, 62–83, 94–104, 127–135, 142–150
Deleage & Roux beta-turn regions	5–9, **16–26,** 28–32, 55–63, 84–89, 114–118

Notes: The possible predominant B epitope region showing conformity with the result of phage-displayed peptide library is shown in bold.

### Properties analysis of synthetic peptide

**Figure 3:**
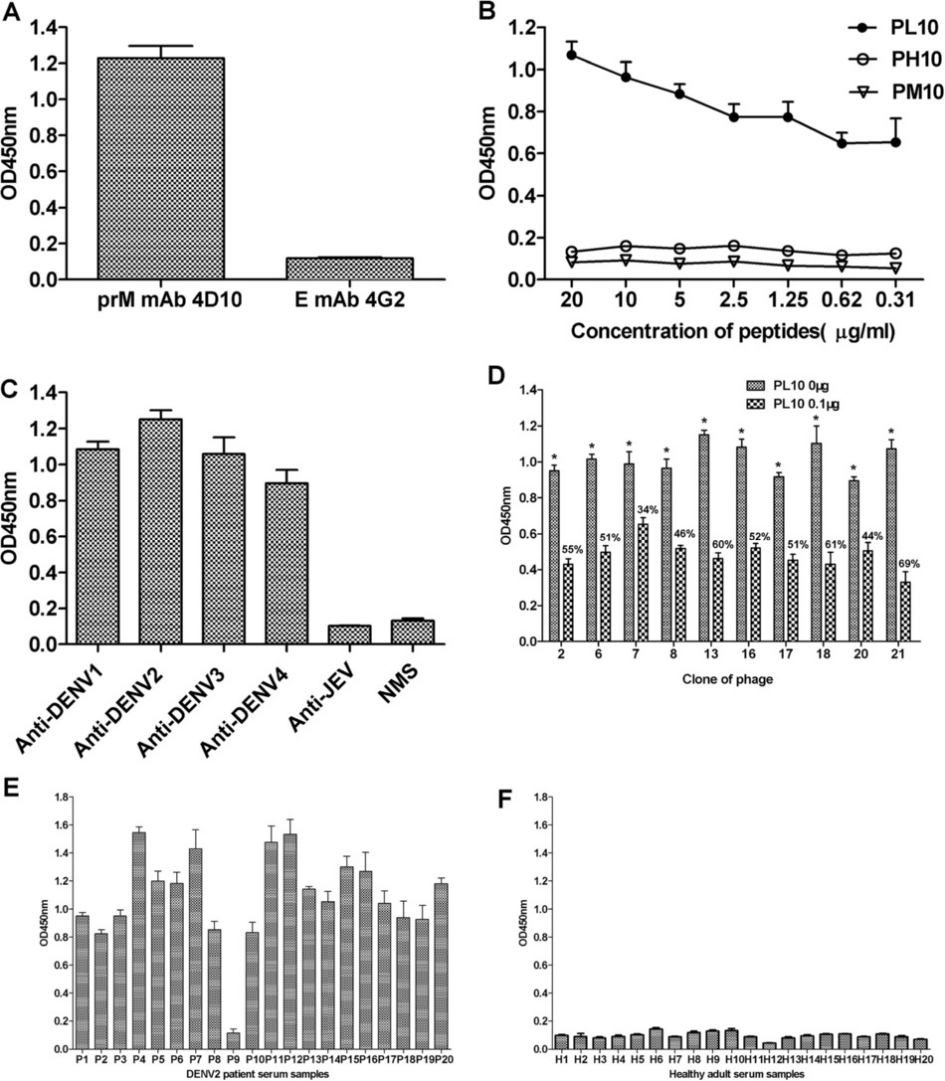
**Properties analysis of synthetic peptide PL10. (A)** Specific reactivity of PL10 with antibody 4D10 (anti-DENV1-4 prM mAb). The synthetic peptide PL10 could react with mAb 4D10 but control antibody 4G2 (anti-flavivirus E mAb) could not. **(B)** The sensitivity binding assay of synthetic peptides PL10 and two control peptides (PH10 and PM10) with mAb 4D10. The synthetic peptide PL10 bound the antibody in a concentration-dependent manner, but two control peptides had no reactivity with 4D10. **(C)** ELISA reactivities of synthetic peptide PL10 with immunized mice sera. Synthetic peptide PL10 was recognized by anti-DENV1-4 mice sera, whereas it was not recognized by anti-JEV mice sera and normal mice sera (NMS). **(D)** Competitive inhibition of phage clone binding to mAb 4D10 by synthetic peptide PL10. Competitive ELISA was performed using PL10 as competitor of its corresponding phage clones. The percentage of inhibition is also shown. **(E** and **F)** ELISA reactivities of synthetic peptide PL10 with serum samples from 20 DENV2-infected patients **(E)** and 20 healthy adults **(F)**. PH10 and PM10 were used as control. Data are expressed as means of at least three independent experiments. The error bars represent standard deviations (SD). If there is no error bar, it is not that no variations among three independent experiments but that the variations are too small to show in the figure. * *P* < 0.05 vs PL10 at 0.1 μg.

To verify that peptide sequence corresponding to amino acid residuals 14–18 of the prM protein of DENV was indeed recognized by the antibody 4D10, synthetic peptide PL10 (^13^IVSRQEKGKS^22^) binding assays were carried out. As shown in Figure 3A, 4D10 specifically reacted with the synthetic peptide PL10, whereas control antibody 4G2 (anti-flavivirus E mAb) did not reacted with PL10. For the sensitivity binding assay, the synthetic peptide PL10 bound the antibody in a concentration-dependent manner. Two control peptides PH10 (^3^LTTRGGEPHM^12^) and PM10 (SQNPPHRHQS) were not reactive (Figure 3B).

We next evaluated whether the synthetic peptide PL10 could be react with anti-DENV1-4 mice sera. Synthetic peptide PL10 was recognized by anti-DENV1-4 mice sera, whereas it was not recognized by anti-JEV mice sera and normal mice sera (NMS) (Figure 3C). We concluded that synthetic peptide PL10 is a DENV serocomplex cross-reactive epitope-based peptide.

To confirm further the phage-displayed peptide was the epitope of antibody 4D10, a peptide competitive-inhibition assay was performed to determine whether the PL10 peptide competed with corresponding phage clones for reactivity with 4D10. The reaction activity of antibody 4D10 with the corresponding phage clones was inhibited markedly by PL10 at 0.1 μg per well with the inhibition percentage from 34% to 69% (Figure 3D). The results showed that the synthetic peptide and corresponding phage clones competed for the same antibody-binding site. Together, these findings suggest that 4D10 recognizes a new epitope on the N-terminal segment of DENV1-4 prM protein.

Then, we evaluated the reactivity of synthetic peptide PL10 with DENV2 patient serum samples. PL10 was able to detect 19 serum samples obtained from 20 DENV2 patients (Figure 3E). The sensitivity of PL10 serologic test was 95%. In addition, the level of antibody varied among patients. In contrast, all of the serum samples collected from 20 healthy adults were shown to be seronegative (Figure 3F).

### Immunogenicity of synthetic peptide on Balb/c mice

**Figure 4:**
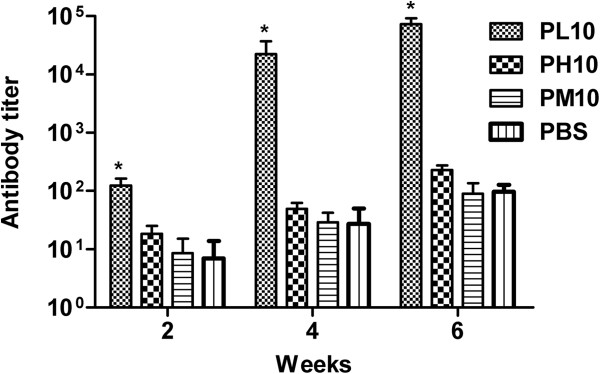
**Time course of antibody titer levels induced in mice immunized with PL10, PH10, PM10 and PBS.** Four groups (PL10 coupled to KLH, PH10 coupled to KLH, PM10 coupled to KLH, and PBS), each comprising of ten adult female Balb/c mice (4–6weeks old), were immunized thrice with 2-week intervals using 100 μg of the PL10, PH10, PM10 or an equal volume PBS. PH10 ,PM10 and PBS were used as control. The mice were bled on week2, 4 and 6 via tail vein, and the anti-peptide antibody titer of mice sera was determined by ELISA. The antibody titer of PL10 was remarkably higher than that of the antisera from mice immunized with PH10, PM10 and PBS at each time point. Data are expressed as means of at least three independent experiments. The error bars represent standard deviations (SD); * *P* < 0.05 vs control groups (PH10, PM10 and PBS).

The antibody titer values were measured after immunization of Balb/c mice with PL10 coupled to KLH, PH10 coupled to KLH, PM10 coupled to KLH or PBS. Sera of preimmunization group were tested at 1:100 dilution to yield the values of background. The antibody titer of sera from mice immunized with PL10 was remarkably higher than that of the sera from mice immunized with PH10, PM10 and PBS (P <0.05). And there was a significant increase of antibody titer of PL10 after the second boost immunization (Figure 4).

### Protection response in adult Balb/c mice

**Figure 5:**
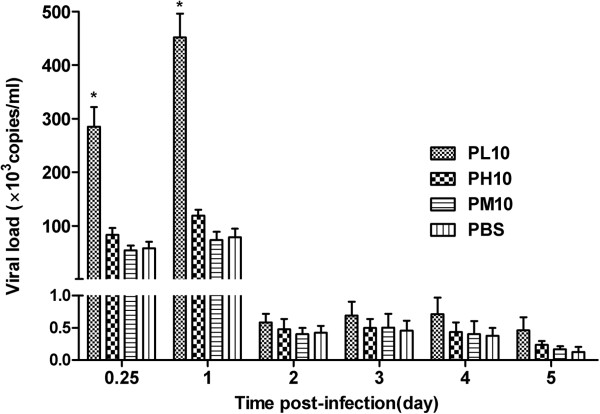
**Quantification of viral RNA levels in immunized mice after inoculated with DENV2 NGC strain.** Two weeks after the last immunization, mice were infected with DENV2 NGC strain through peritoneal injection. Viral RNA levels of sera were quantified by qRT-PCR at day 0.25, 1, 2, 3, 4 and 5 post-infection. PH10 and PM10 were used as control. The vial RNA levers in the PL10 group were always higher than that in groups of PH10, PM10 and PBS at any given time point. Data are expressed as means of at least three independent experiments. The error bars represent standard deviations (SD); * *P* < 0.05 vs control groups (PH10, PM10, PBS).

Two weeks after the last immunization, groups of mice were infected with DENV2 NGC strain (10^6^ PFU/mouse). The viral RNA copy numbers of sera were quantified by qRT-PCR (Figure 5). We detected high levels of viral RNA in all groups at day 0.25 and 1 post-infection, but the viral RNA copies were significantly reduced in all the groups at day 2, 3, 4 and 5 post-infection. In spite of that, the vial RNA levers in the PL10 group were remarkably higher than that in groups of PH10, PM10 and PBS at day 0.25 and 1 post-infection (P <0.05).

### Infectious properties of standard DENV2 and imDENV2

**Figure 6:**
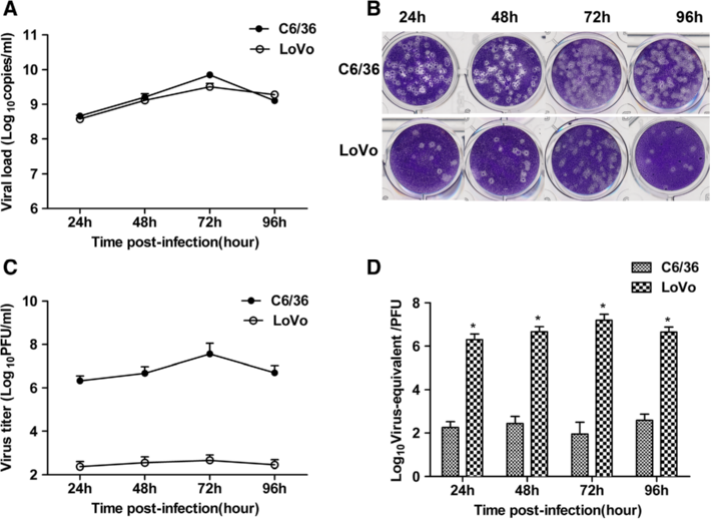
**The infectious properties of standard DENV2 and imDENV2 determined by qRT-PCR and plaque forming assay.** The viral RNA copies determined by qRT-PCR **(A)** and the plaque morphology and infectious titer determined by plaque forming assay **(B** and **C)** of DENV2 produced in C6/36 and LoVo cells at each time point. **(D)** The ratio of viral RNA copies (copies/ml) to infectious titer (PFU) for the distinct virus preparations. The specific infectivity of imDENV2 was significant lower than that of DENV2 generated in C6/36 cells. Data are expressed as means of at least three independent experiments. The error bars represent standard deviations (SD). If there is no error bar, it is not that no variations among three independent experiments but that the variations are too small to show in the figure. * *P* < 0.05 vs C6/36.

The growth and specific infectivity of the viruses were measured by qRT-PCR and plaque forming assay respectively. As shown in Figure 6A, we determined the viral RNA copies by qRT-PCR and found that LoVo and C6/36 cells released comparable viral RNA copies at each time point examined. This indicates that the capacity of releasing viral particles is not impaired in furin-deficient LoVo cells. In both cell lines, we detected maximal virus particles released at 72 hpi. Next, we determined the infectious properties of the distinct virus preparations by plaque forming assay. The infectious titer of imDENV2 was severely reduced than that of virus produced in the C6/36 cells at any given time point (Figure 6B and C). Subsequently, we calculated the ratio of viral RNA copies (copies/ml) to infectious titer (PFU) for each of the virus samples (Figure 6D). The virus-equivalent particles per PFU of LoVo cells was remarkably higher than that of C6/36 cells. These results showed that the specific infectivity of imDENV was at least 10, 000-fold lower compared with that of virus produced in C6/36 cells.

### Plaque reduction neutralization test

**Figure 7:**
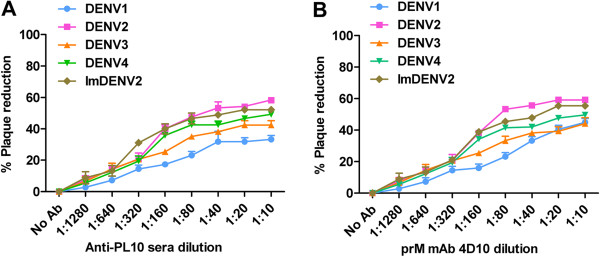
**Partial neutralizing activities of mAb 4D10 and anti-PL10 sera.** Serial 2-fold dilutions of antibody were mixed with approximately 50 PFU DENV and incubated for 1 h at 37°C. Neutralizing activities were evaluated by plaque reduction assay using BHK21 cells. Both 4D10 **(A)** and anti-PL10 sera **(B)** exhibited partial neutralizing activities against standard DENV-4 and imDENV2. Data are expressed as means of at least three independent experiments. The error bars represent standard deviations (SD). If there is no error bar, it is not that no variations among three independent experiments but that the variations are too small to show in the figure.

Neutralizing activities of mAb 4D10 and anti-PL10 sera for standard DENV1-4 and imDENV2 were assessed using a standard plaque reduction neutralization assay. We found that 4D10 and anti-PL10 sera were unable to completely neutralize infection (Figure 7). Instead, neutralization level ranged from 33.3% to 59.2%, and the partial neutralization was cross-reactive among the four virus serotypes. These antibodies did not exhibit a high level of neutralization. Although infectivity of imDENV2 was severely reduced, it remained partially susceptible to neutralization and the titration curve for DENV2 produced in LoVo and C6/36 cells were similar (Figure 7).These results indicate that mAb 4D10 and anti-PL10 sera could not potently neutralize standard DENV1-4 and imDENV2.

### ADE of DENV infection mediated by 4D10 and anti-PL10 sera

**Figure 8:**
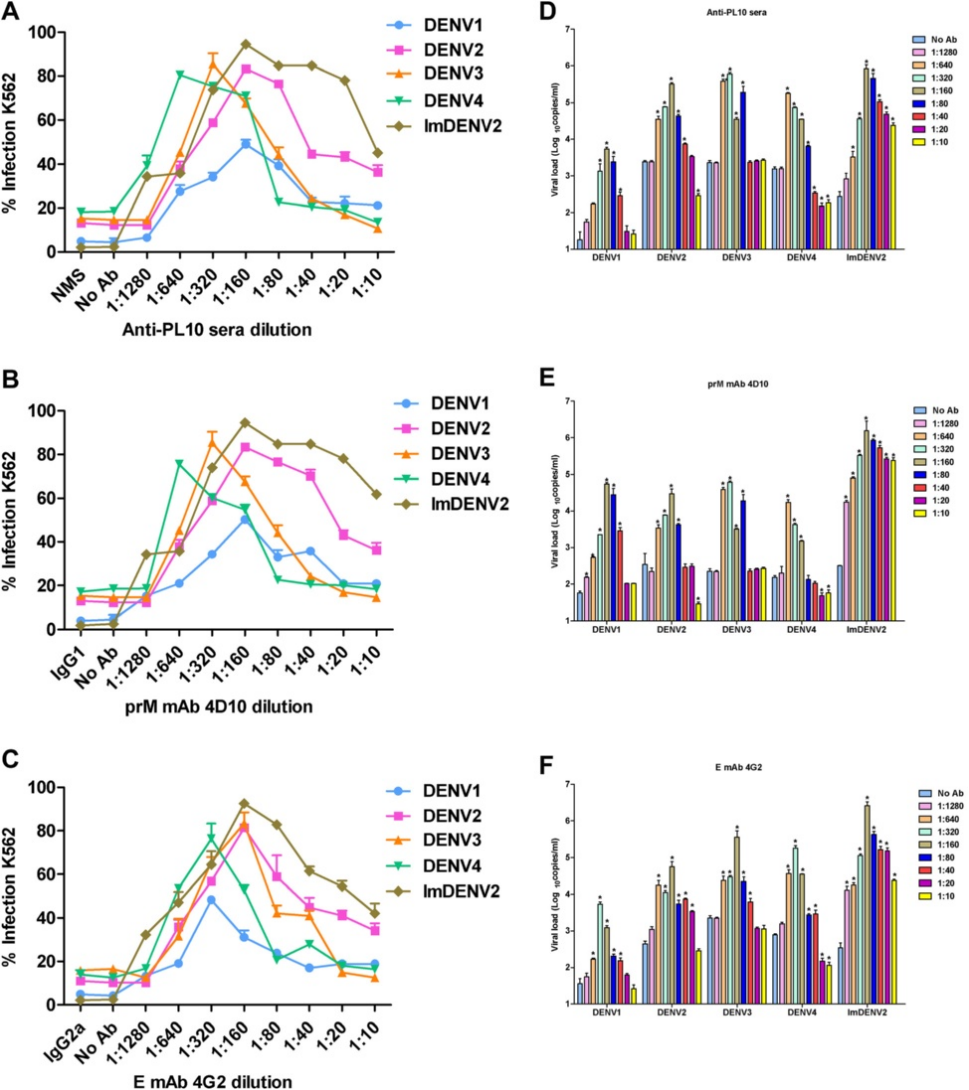
**Infection enhancement of DENV mediated by 4D10, anti-PL10 sera and 4G2 in K562 cells.** Serial 2-fold dilutions of antibody were incubated with an equal volume of DENV for 1 h at 37°C then transferred to K562 cells at MOI of 1. Infected K562 cells were determined by flow cytometry at 3 days post infection **(A, B** and **C)**. Viral RNA copies of infected supernatants were measured by qRT-PCR at 4 days post infection **(D, E** and **F)**. Both 4D10 and anti-PL10 sera could potently enhance infection of standard DENV1-4 and imDENV. NMS and IgG (mouse IgG1 and mouse IgG2a) isotype control antibodies were used as controls. Data are expressed as means of at least three independent experiments. The error bars represent standard deviations (SD). If there is no error bar, it is not that no variations among three independent experiments but that the variations are too small to show in the figure. * *P* < 0.05vs No Ab.

We carried out ADE assays with Fc receptor-bearing K562 cells to determine the enhancing capacity of 4D10, anti-PL10 sera and 4G2 (positive control) towards standard DENV1-4 and imDENV2 using flow cytometry and qRT-PCR. Previous studies have shown that 4G2 was capable of enhancing infection of standard DENV1-4 and imDENV2 [24,54]. Consistent with these reports, enhancement of infection was observed for 4G2 in this study (Figure 8C and F). According to flow cytometry results, enhancement of infection was observed for 4D10 and anti-PL10 sera with a peak of nearly 80% increase (Figure 8A and B), the enhanced infection percentage varied from 2.2% to 79.3% over a large range of antibody concentration among four DENV serotypes. Next we tested enhancement of imDENV2 using a constant amount of virus-equivalent particles compared to DENV2. The results showed that the normally non-infectious imDENV2 could be rendered much more infectious in the presence of 4D10 and anti-PL10 sera than DENV2 did (Figure 8A and B). These results were further exemplified by assessing viral RNA copies in infected supernatants using qRT-PCR (Figure 8D and E). Consistent with flow cytometry results, 4D10 and anti-PL10 sera led to a significant increase of viral load over a broad antibody concentration range (P <0.05). Taken together, both 4D10 and anti-PL10 sera could potently enhance infection of standard DENV and imDENV2.

## Discussion

It has been previously reported that anti-prM mAbs provided cross-protection against all four DENV serotypes [40,55]. However, the potential importance of these prM-containing particles and prM antibodies in DENV infection pathogenesis has been ignored for a long time as numerous functional studies have reported that fully immature particles are noninfectious [56]. Interestingly, recent studies on human and mouse anti-prM mAbs [24-32] suggest that prM-specific mAbs have a significant role to enhance infection of standard DENV and imDENV particles. However, there have been few attempts to locate the epitopes of prM ptotein. To gain a deeper understanding of the antigenic structures of prM and their functions in human immune response to DENV, we identified the epitope of prM mAb 4D10 and investigated the ability of mAb 4D10 and antibody against epitope peptide PL10 to mediate ADE infection of standard DENV1-4 and imDENV particles.

In this study, we generated and characterized a DENV serocomplex cross-reactive prM mAb 4D10. Then, we successfully mapped the epitope of 4D10 to amino acid residues 14 to 18 of DENV1-4 prM protein using phage display technology. The epitope peptide showed conformity with one region (amino acid residues 12 to 26) predicted by bioinformatics analysis. Consequently, the epitope peptide (^13^IVSRQEKGKS^22^) was synthesized for further study. We confirmed that PL10 was a DENV serocomplex cross-reactive epitope peptide and showed to be highly immunogenic in Balb/c mice. Also, PL10 could successfully distinguish DENV serotypes from other flaviviruses in immunized mice sera. The high degree of antibody cross-reactivity among different flaviviruses has been a diagnostic challenge to distinguish various flaviviral infections, and this limitation is apparent for members of DENV serotypes [57,58]. It has been previously reported that prM-specific antibodies could be applied as a diagnostic marker to distinguish previous infection of DENV from JEV [22]. Thus, it is remarkable that the DENV-specific epitope in prM has great potential to improve DENV serological diagnostic tests. Furthermore, PL10 could successfully recat with DENV2-infected patient sera but not with sera of healthy donors, suggesting that the epitope peptide PL10 could possibly be used as a serologic reagent in the diagnosis of DENV-infected patients.

The control peptide PH10 (^3^LTTRGGEPHM^12^) may be the possible epitope region of prM protein predicted by bioinformatics analysis, but the antibody titer of PH10 was not high enough. For synthetic peptides to serve as effective immunogens, they must comprise potential antigenic sites to promote B cell interaction [59].

Immature particles produced in furin-deficient LoVo cells have very high levels (94%) of prM-containing particles. Interestingly, both mammalian cells (BHK-21 or Vero) and insect cells (C6/36) infected with DENV release as many as 30% prM- containing immature particles [42,60] suggesting that cleavage of prM to M is not very effective. Therefore, cells infected with DENV release a heterogeneous mixture of not only fully mature(containing M) and immature (containing prM) but also partially mature virus particles (containing prM and M) [42,61,62]. Previous studies have demonstrated that mature particles and partially mature particles were infectious whereas immature particles were virtually non-infectious [24,27,42,56]. The maturation state of virus particles can influence the neutralizing and enhancing capacity of antibodies direct against DENV surface proteins [24,27,63]. We detected the specific infectivity of the LoVo-released virus particles and found that the infectious properties of imDENV2 was 10,000-fold lower compared to that of C6/36-cultured standard virus preparations. This agrees with previous results [27,42] and proves that immature virus is virtually non-infectious.

Antibodies induced by DENV infection may have dual roles: obstruct infection through neutralization activity or enhance viral infection via ADE activity. Consistent with prior studies [24-27,31,41,42], the mAb 4D10 and antibody against epitope peptide PL10 described in the present study showed broad cross-reactivity and poor neutralizing activity with the four standard DENV serotypes and imDENV but significantly enhanced the infectious properties. These results suggested 4D10 and anti-PL10 sera were infection-enhancing antibodies and PL10 was infection-enhancing epitope. We found mAb 4D10 and antibody against PL10 showed different neutralizing against different virus strains, suggesting the existence of structural differences in the epitope region. The mechanism of virus neutralization and ADE in the presence of antibody against prM is still elusive. Consistent with these results, during protection assay in vivo, our data clearly suggested the epitope peptide PL10 indeed elicit enhancing antibodies and promote DENV replication. The partial neutralization of antibodies against prM to standard dengue viruses implies that some infectious particles within the virus preparation are partially mature (containing a mixture of prM and M) and also indicates that prM antibodies have the capacity to block the infectivity of partially mature particles. Meanwhile, partial cleavage of prM from the viral surface reduces available antigens for neutralization activity. The cross-reactive among four DENV serotypes, together with partial cleavage of prM, makes dengue viruses susceptible to ADE by antibody against prM [24,56]. It was recently shown that anti-prM antibodies could render essentially non-infectious imDENV particles highly infectious. The prM antibodies bind to the virion surface prM antigens and facilitate efficient binding and cell entry of virus-antibody complexes into Fc receptor-bearing cells following which the endosomal furin clears prM into M and renders immature particles infectious [24,27]. Taken together, our results support the notion that antibodies against prM can enhance infectivity of prM-containing immature and partially mature DENV particles due to an interaction with Fc receptor expressed on immune cells.

As shown in Figure 8A and B, at low dilution of antibody, enhancing and neutralizing activities are mixed and enhancement is suboptimal, so little or no infection enhancement is found. Then, enhanced viral growth occurs at a higher dilution. At some dilution of antibody, optimal viral infections occur and peak enhancement is observed. At a still higher dilution, the concentration of infectious antibody–virus complexes is not great enough to elicit the system response and the infection enhancement is gradually lost [64]. The peak infection enhancement also need a large number of virus receptors on FcR-bearing cells, the efficient cell entry of virus, the viability of virus in the cytosol, and capability to accomplish all steps to achieve assembly and final release of virus particles.

Since recent studies found that DENV particles released from infected cells contained as many as 30% prM particles, the infectious potential of immature particles may have significant implications for understanding of the dengue pathogenesis. In the early stages of a primary infection, immature particles fail to enter host cells in the absence of antibodies, and therefore are of minor importance in disease development. On the other hand, prM-specific antibody response will activate the infectivity of fully immature particle upon secondary infection, and increase the number of infectious particles.

The epitope recognized by our own anti-prM antibody was located in amino acid residuals 14–18 of the prM protein and was different from the published sequence recognized by other anti-prM mAb 2H2 (mapped to amino acid residuals 40–49) and 70-21 (mapped to amino acid residuals 53–67) [40,41]. Previous studies have shown that 2H2 provided cross-protection against all four DENV serotypes [40,55]. However, many studies demonstrated that 2H2 could enhance the infectivity of standard DENVV and imDENV [27,65,66]. Also, antibody 70–21 as well as many other prM mAbs has been reported to enhance DENV infectivity [24,26,27,31]. Our results support that anti-prM antibodies could enhance infectious properties of DENV and prM epitopes could be not protective but infection enhancing. We propose that the length of epitope sequence has an important role to mediate ADE infection. For long epitope peptide sequences, they may contain two or more epitopes, which may be immunodominant or cryptic. These findings suggest that antigenic structures of prM and their functions are complicated and not well studied.

Most current dengue vaccines contain native dengue prM, it may be important to consider better vaccine approaches that eliminate ADE activities induced by infection-enhancing epitopes on prM during vaccine design [24]. Vaccine candidates that eliminate pathogenic infection-enhancing epitopes may thus become increasingly important. Most importantly, identification of the epitopes on prM protein will provide new insights for further understanding of humoral immune responses to DENV at the epitope level. However, to our knowledge, there have been few reports of epitopes mapping to aim at prM protein. Here, we report a novel infection-enhancing epitope on dengue prM, the findings from our study may have significant implications for future vaccine design and facilitate understanding the pathogenesis of DENV infection.

## Conclusions

We mapped the epitope of 4D10 to amino acid residues 14 to 18 of DENV1-4 prM using a phage-displayed peptide library and comprehensive bioinformatic analysis. Then, we found that this epitope was infection-enhancing. These findings may provide important information for the understanding of the pathogenesis of DENV infection at epitope level and contribute to the development of dengue vaccine.

## Abbreviations

DENV: Dengue virus; ADE: Antibody-dependent enhancement; prM: Pre-membrane; mAb: Monoclonal antibody; imDENV: Immature DENV; DF: Dengue fever; DHF: Dengue hemorrhagic fever; DSS: Dengue shock syndrome; NS: Nonstructural; TGN: Trans-Golgi network; FBS: Fetal bovine serum; BHK-21: Baby hamster kidney-21; NGC: New Guinea C; MOI: Multiplicity of infection; Hpi: Hours post-infection; qRT-PCR: Real-time quantitative RT-PCR; ELISA: Enzyme-linked immunosorbent assay; IFA: Indirect immunofluorescence assay; PFU: Plaque-forming units; HRP: Horseradish-peroxidase; CO2: Carbon dioxide; ECL: Enhanced chemiluminescence reagents; PRNT: Plaque reduction neutralization test; NMS: Normal mouse serum.

## Competing interests

The authors declare that there have no competing interests.

## Authors’ contributions

LFJ and YYL designed the experiments. YYL carried out most of the experiments and wrote the manuscript. JMZ helped to analysis and interpretation of data. JJF and ZJY participated in animal experiments. DYF carried out virus isolation and multiplication. LFJ revised the manuscript. HJY and GCZ participated in part of experiments. All authors read and approved the final manuscript.
